# *Methanomethylovorans* are the dominant dimethylsulfide-degrading methanogens in gravel and sandy river sediment microcosms

**DOI:** 10.1186/s40793-024-00591-4

**Published:** 2024-07-20

**Authors:** S. L. Tsola, A. A. Prevodnik, L. F. Sinclair, I. A. Sanders, C. K. Economou, Ö. Eyice

**Affiliations:** School of Biological and Behavioural Sciences, Queen Mary University of London, London, UK

**Keywords:** Dimethylsulfide, Methylotrophic methanogenesis, River sediments, Metagenomics

## Abstract

**Background:**

Rivers and streams are important components of the global carbon cycle and methane budget. However, our understanding of the microbial diversity and the metabolic pathways underpinning methylotrophic methane production in river sediments is limited. Dimethylsulfide is an important methylated compound, found in freshwater sediments. Yet, the magnitude of DMS-dependent methanogenesis nor the methanogens carrying out this process in river sediments have been explored before. This study addressed this knowledge gap in DMS-dependent methanogenesis in gravel and sandy river sediments.

**Results:**

Significant methane production via DMS degradation was found in all sediment  microcosms. Sandy, less permeable river sediments had higher methane yields (83 and 92%) than gravel, permeable sediments (40 and 48%). There was no significant difference between the methanogen diversity in DMS-amended gravel and sandy sediment microcosms, which *Methanomethylovorans* dominated. Metagenomics data analysis also showed the dominance of *Methanomethylovorans* and *Methanosarcina*. DMS-specific methyltransferase genes (*mts*) were found in very low relative abundances whilst the methanol-, trimethylamine- and dimethylamine-specific methyltransferase genes (*mtaA*, *mttB* and *mtbB*) had the highest relative abundances, suggesting their involvement in DMS-dependent methanogenesis.

**Conclusions:**

This is the first study demonstrating a significant potential for DMS-dependent methanogenesis in river sediments with contrasting geologies. *Methanomethylovorans* were the dominant methylotrophic methanogen in all river sediment microcosms. Methyltransferases specific to methylotrophic substrates other than DMS are likely key enzymes in DMS-dependent methanogenesis, highlighting their versatility and importance in the methane cycle in freshwater sediments, which would warrant further study.

**Supplementary Information:**

The online version contains supplementary material available at 10.1186/s40793-024-00591-4.

## Introduction

Freshwater ecosystems such as lakes, rivers and streams are important components of the global carbon cycle. These ecosystems account for around half of the methane emitted to the atmosphere (538–884 Tg y^−1^), contributing considerably to the global greenhouse gas budget [53]. In particular, rivers were estimated to emit 27.9 Tg methane per year despite accounting for around 0.58% of the Earth’s non-glaciated surface area [1, 52]. Since the importance of rivers for global methane emissions has only recently been acknowledged, studies on methane production pathways and underlying microbial populations in the river sediments are scarce, impeding progress in projecting global greenhouse gas emissions.

Recent studies suggest a strong influence of climatic factors, adjacent soil characteristics (e.g. organic carbon stock, groundwater table depth and primary production) and geomorphological variables (e.g. sediment properties, river slope and elevation) on methane production and emission rates from river ecosystems [52]. Among the physical factors affecting methane production is the sediment grain size, which may substantially alter the sediment permeability, organic and inorganic matter content and sediment oxygen concentrations [19, 22, 26, 37, 52]. For instance, sandy riverbeds are less permeable than gravel riverbeds and are most likely to contain anoxic zones, where methane production can be observed [3, 24, 31, 59, 66].

Methanogenesis in river sediments has mainly been attributed to the hydrogenotrophic and acetoclastic pathways; however, methanogenesis through the degradation of methylated compounds such as methanol and dimethylsulfide (DMS) was also shown in freshwater ecosystems [38, 38, 39, 39, 40]. In freshwater sediments, the degradation of sulfur-containing aminoacids and methoxylated aromatic compounds as well as methanethiol methylation and dimethyl sulfoxide (DMSO) reduction results in DMS production [55, 56]. However, DMS degradation was only studied in freshwater peatland and lake sediments [15, 38, 39]. More recently, we have demonstrated that as much as 41% of DMS amended to the River Medway (UK) sediments were anaerobically degraded to methane, likely contributing to the in situ methane production in this river sediment [68].

Methanogen populations undertaking anaerobic DMS degradation and their metabolic pathways are poorly characterised. A limited number of species from the genera *Methanomethylovorans*, *Methanolobus*, *Methanosarcina* and *Methanohalophilus* were isolated from freshwater and saline ecosystems as DMS-degrading methanogens [16, 25, 36, 38, 39, 42, 45, 47]. Further, methylthiol:coenzyme M methyltransferase (Mts) was shown to catalyse the formation of methane from DMS in *Methanosarcina* strains [18, 46, 63]. Our recent study has also shown the involvement of methanol- and trimethylamine-methyltransferases in anaerobic DMS degradation in brackish sediments [67]. Yet, we do not know whether these represent the dominant DMS-degrading methanogen populations and key genes in this process in river sediments.

We hypothesised that DMS may be an important methane precursor in river sediments, which may harbour different methylotrophic methanogen populations based on grain size and permeability. We tested this hypothesis by quantifying the extent of DMS-dependent methanogenesis in river sediments with contrasting grain sizes and characterising the methanogen diversity driving this process. We also provided the first insight into the potential metabolic pathways of DMS-dependent methane production in river sediments.

## Methods

### Site characteristics and sediment sampling

**Fig. 1:**
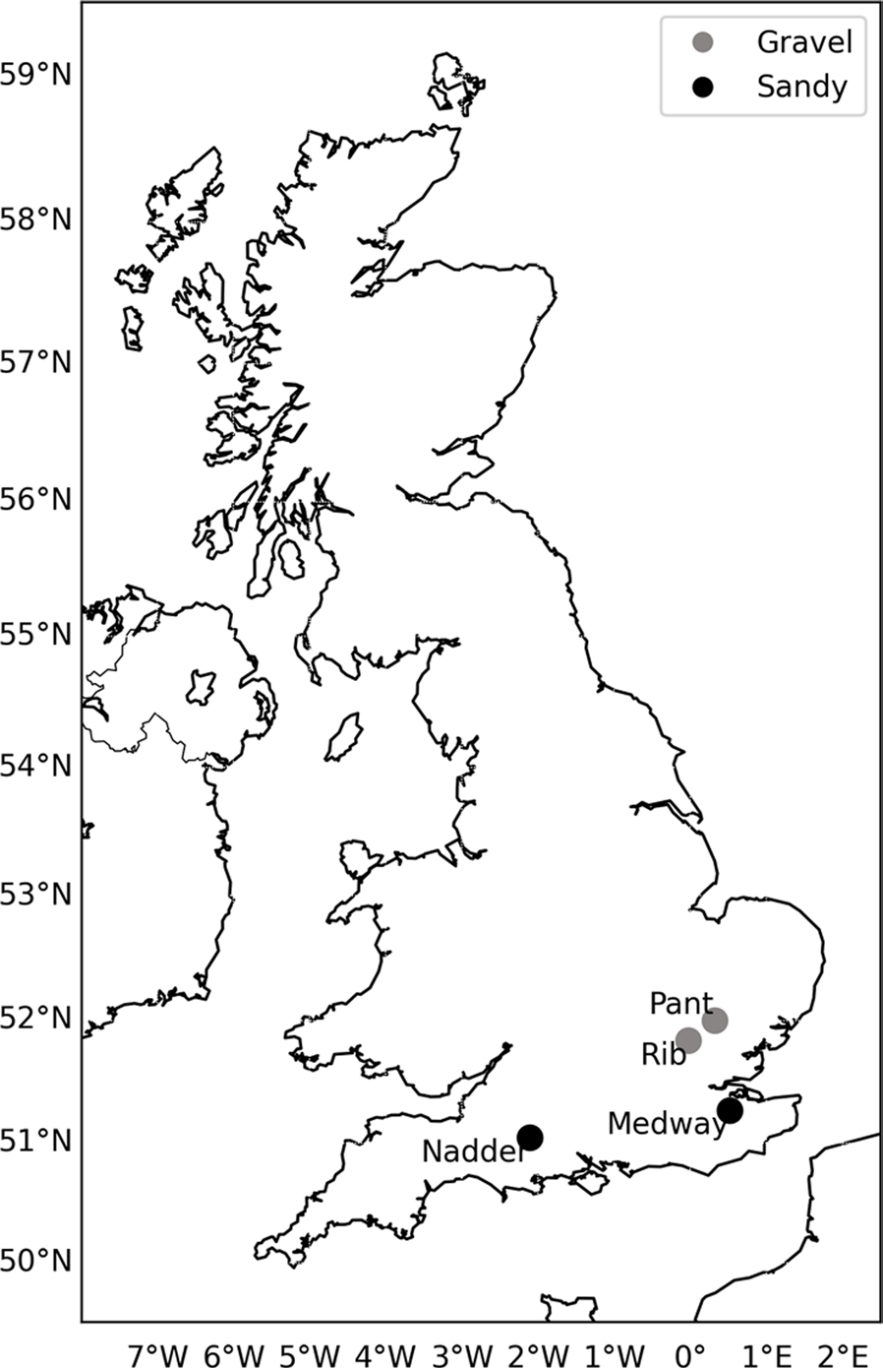
The UK map and the location of the rivers sampled for this study. Colour indicates sediment grainsize. Light grey: Gravel, black: Sandy. The coordinates for the rivers are: River Pant—52.0044, 0.316916; River Rib—51.83917, − 0.02936; River Medway—51.26798, 0.518439; River Nadder—51.04385, − 2.11182

Three sediment cores (3.5 cm Ø) were collected from each sampling site in the UK in February 2019 (Rivers Pant and Rib) and in March 2021 (Rivers Medway and Nadder; Fig. 1) to set up microcosms. Approximately 1 L of sediment from the top 5 cm were also collected from the sites and were screened through 2 mm and 0.0625 mm sieves to characterise the sediment as gravel (sediment size > 2 mm) or sandy (2 mm < sediment size < 0.0625 mm; Supplementary Table 1; [73]).

### Microcosm set-up

Five replicated microcosms were set up per sampling site in 140 mL serum bottles (Wheaton, USA) in an anaerobic glove box (Belle Technology, UK). We mixed the sediment from the three cores (depth 4–10 cm) and used 5 g of homogenised sediment and 40 mL of growth medium (pH 7), which contained 3.2 mM NaCl, 0.1 mM MgSO_4_·7H_2_O, 0.088 mM NaNO_3_, 0.031 mM CaCl_2_·2H_2_O, 0.1 mM MgCl_2_·6H_2_O, 0.9 mM Trizma base, 2.1 µM K_2_HPO_4_^**.**^3H_2_O, trace elements, and vitamins [76, 77]. The trace element solution was prepared according to DMSZ media 141 and modified to contain FeC_6_H_6_O_7_ (5 g) instead of N(CH_2_CO_2_H)_3_ and no Na_2_WO_4_·2 H_2_O.

Samples were initially amended with 2 µmol DMS g^−1^ wet sediment through gas-tight glass syringes as the only energy and carbon source. After the first DMS addition was depleted, 4 µmol DMS g^−1^ wet sediment was added in each subsequent amendment to avoid DMS toxicity. A total of 43–154 µmol DMS g^−1^ sediment was consumed by the end of the incubation period. Two sets of controls (three replicates per each set) were also established. One set contained no DMS and the second set contained thrice autoclaved sediment with DMS to monitor the DMS adsorption by sediment. All microcosm bottles were kept at 22 °C and in the dark to avoid photochemical destruction [7].

### Analytical measurements

Headspace DMS in the microcosm bottles was measured using a gas chromatograph (GC; Agilent Technologies, 6890A Series, USA) fitted with a flame photometric detector (FPD) and a J&W DB-1 column (30 m × 0.32 mm Ø; Agilent Technologies, USA). The oven temperature was set at 180 °C, and zero grade N_2_ (BOC, UK) was used as the carrier gas (26.7 mL min^−1^). FPD run at 250 °C with H_2_ and air (BOC, UK) at a flow rate of 40 and 60 mL min^−1^, respectively. DMS standards were prepared by diluting > 99% DMS (Sigma-Aldrich, USA) in distilled water previously made anaerobic with oxygen-free N_2_ (BOC, UK).

Methane and carbon dioxide (CO_2_) were measured using a GC (Agilent Technologies, USA, 6890N Series) fitted with a flame ionisation detector (FID), Porapak (Q 80/100) packed stainless steel column (1.83 m × 3.18 mm Ø; Supelco, USA), and hot-nickel catalyst, which reduced CO_2_ to methane (Agilent Technologies, USA; [54]). The oven temperature was set at 30 °C, and zero grade N_2_ (BOC, UK) was used as the carrier gas (14 mL min^−1^). FID run at 300 °C with H_2_ and air (BOC, UK) at a flow rate of 40 and 430 mL min^−1^, respectively. Certified gas mixture was used as standards (100 ppm methane, 3700 ppm CO_2_, 100 ppm N_2_O, balance N_2_; BOC, UK). All methane concentrations were corrected for the headspace-water partitioning using Henry’s law to calculate the total methane.

The total production of CO_2_ was the sum of the change of CO_2_ in the headspace and the total dissolved inorganic carbon (ΣDIC = CO_2_ + HCO_3_^−^ + CO_3_^2−^) in the water phase. For the ΣDIC, 3 mL of supernatant from each microcosm was collected in 3 mL gas-tight vials (Exetainer, Labco, UK) and fixed using 24 µL ZnCl_2_ (50% w/v). 100 µL of 35% HCl was added to acidify the samples and CO_2_ in the headspace was measured. An inorganic calibration series (0.1–8 mM; Sigma-Aldrich, USA) of Na_2_CO_3_ was used as standard [58].

Methane and CO_2_ concentrations in the control bottles were between 0.45–20 µmol/g and 0.035–1.95 µmol/g, respectively. These values were subtracted from the concentrations measured in DMS-amended microcosms.

### Sequencing library preparation and sequence data analysis

DNA was collected from the bottles at the end of the incubation period and was extracted from 0.25 g sediment using the DNeasy Powersoil kit (Qiagen, NL) following the manufacturer’s instructions.

The *mcrA* gene, which encodes the *α*-subunit of the methylcoenzyme M reductase from all known methanogens, was used as the methanogen molecular marker. The *mcrA* gene in each replicated microcosm was first amplified using the mcrIRD primer set [32] and a second PCR was carried out using the same primer set with overhang adapters as was described in Tsola et al. [68]. PCR products were further amplified to add dual indices and Illumina sequencing adapters. All PCR products were cleaned using JetSeq Clean beads (1.4x; Meridian Bioscience, USA), normalized using the SequalPrep Normalization Plate kit (Invitrogen, USA) and sequenced at 2 × 300 bp Illumina MiSeq Next Generation sequencing platform.

The sequence analysis was performed using QIIME2 2021.11 on Queen Mary’s Apocrita HPC facility, supported by QMUL Research-IT [5, 30]. Taxonomy was assigned to the ASVs using Naive Bayes classifiers, trained using the feature-classifier command in QIIME2 [4] and a custom-made *mcrA* database. These databases were prepared using FunGenes [17], Python 3.10.8 and the RESCRIPt [50] package in QIIME2 2021.11 [5].

### Quantitative polymerase chain reaction (qPCR)

Quantitative PCR (qPCR) of the *mcrA* gene was performed using the primers mlas-mod-F and mcrA-rev-R [2, 62]. Each qPCR reaction was set up in triplicate using a Mosquito HV (SPT Labtech, UK) and performed as described in Tsola et al. [68]. Reactions contained 0.5 µL gDNA (normalised to 3 ng/µL), 0.1 µL of each primer (10 µM), 2.5 µL SensiFAST SYBR (No-ROX,Meridian Bioscience, USA) and 1.8 µL ultra-pure water. A melt curve analysis was performed to detect non-specific DNA products by increasing the temperature from 65 to 95 °C in 0.5 °C increments. Standard curves were produced using a serial 10-fold dilution of clones containing the *mcrA* gene. The efficiency of all reactions was between 90 and 110%, and the R^2^ value for the standard curves was > 99%.

### Statistical analysis

Statistical analysis of the sequencing data and the principal coordinate analyses (PCoA) with Bray–Curtis dissimilarity were performed using the R package microeco [35]. Spearman’s correlation analyses (r_s_) between the first three PCoA coordinates and experimental variables, such as DMS consumption, methane and CO_2_ production, and grain size, were conducted using PAST (4.2; [20]). Data were visualised using R (4.2.1) on RStudio (2022.07.1; [49]) and ggplot2 [74].

### Shotgun metagenomics

Shotgun metagenomics analysis of the sediment sample from the River Pant microcosm was conducted by the U.S. Department of Energy (DOE) Joint Genome Institute (JGI) using the Illumina NovaSeq 6000 platform (2 × 150 bp). The DNA sample had a concentration of 10 ng/µL as measured by Qubit 2.0 Fluorometer (Invitrogen, USA), an A_260/280_ ratio of 1.75 and an A_260/230_ ratio of 1.88. The sequence analysis was carried out following a well-established JGI-created workflow [13].

The MetaCyc and KEGG databases were used to find the genes associated with methylotrophic methanogenesis and other key genes in methanogenesis (Supplementary Table 2; [9, 27]). The gene counts in the metagenome dataset were normalised using the CPM (copies per million) normalisation method [51]. The CPM values were then log-transformed and shown in a heatmap using R (4.2.1) on RStudio (2022.07.1) and ggplot2 [49, 74].

The genes associated with methylotrophic methanogenesis were further explored for their taxonomic affiliation and grouped according to their genera. This was achieved ﻿using the JGI analysis output and confirmed by the nucleotide Basic Local Alignment Search Tool (BLAST; [8]).

Metagenome-assembled genomes (MAGs) were recovered using MetaBAT 2.12.1 [28]. CheckM 1.0.12 was used for genome completion and contamination estimates [48]. The MAGs were characterised as high (HQ) or medium quality (MQ) according to the Minimum Information about Metagenome-Assembled Genome (MIMAG) standards [6]. The MAGs, which do not contain all rRNA genes were classified as medium-quality. Taxonomic affiliation was inferred using the Integrated Microbial Genome (IMG) and GTDB-tk (0.2.2) databases [11, 12].

## Results

### DMS-dependent methane production in sediments with different grain sizes

Sediment grain size analysis showed Rivers Pant and Rib had permeable gravel riverbed sediments (gravel fraction: 66.7 and 59.8%, respectively), whereas Rivers Medway and Nadder had less permeable sandy sediments (sand fraction: 86 and 90.1%, respectively).

**Fig. 2:**
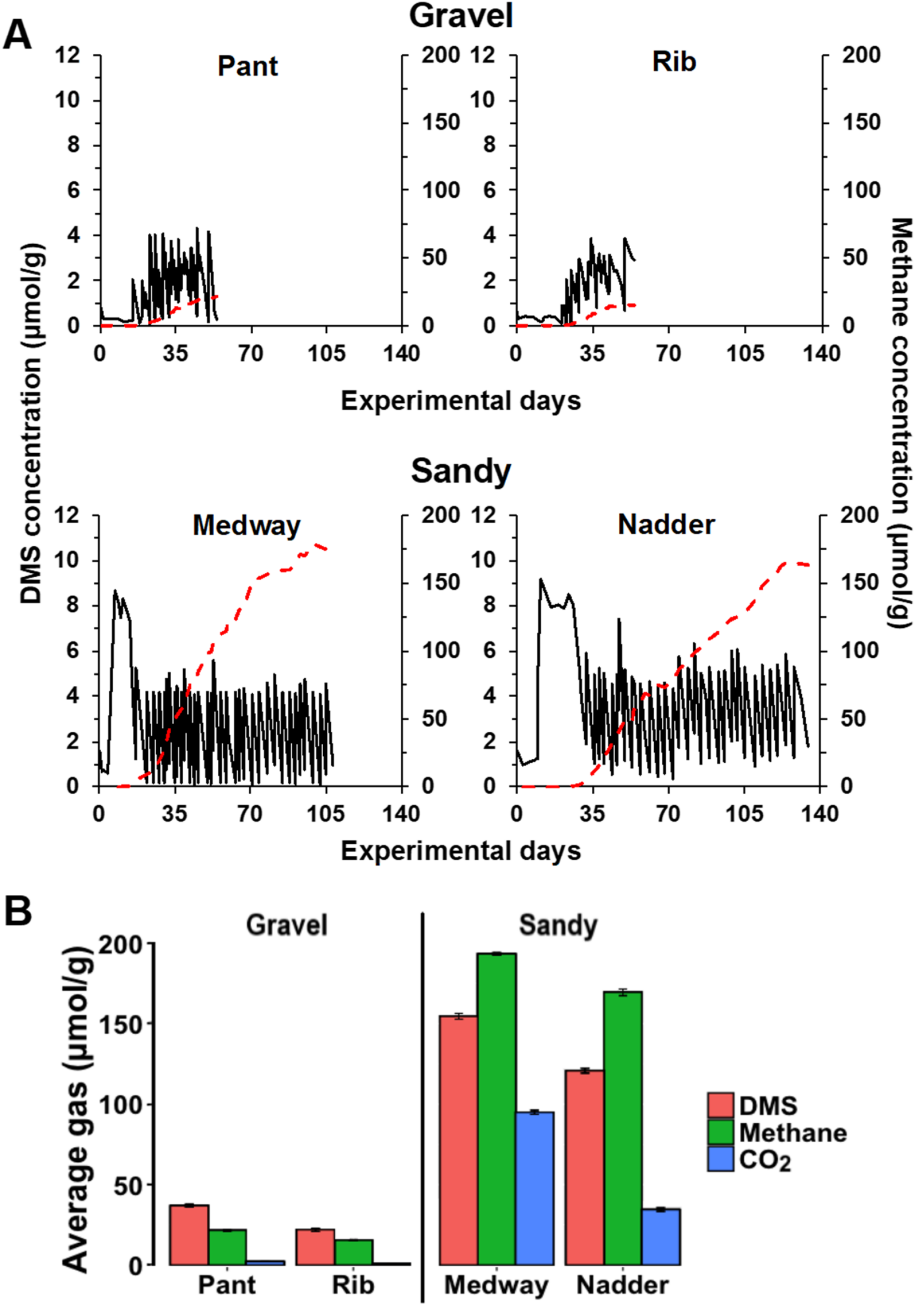
**A** Average DMS and methane concentrations in river sediment microcosms using DMS as the only energy and carbon source. Rivers Pant and Rib have gravel-dominated riverbeds, whereas Rivers Medway and Nadder have sand-dominated riverbeds. Error bars were omitted to make the graph legible. Black line: DMS concentrations, red line: Methane concentrations. **B** Total concentrations of DMS degraded, and methane and CO_2_ produced at the end of the incubation period. Error bars represent standard error above and below the average of five replicates

Methane production was observed in all DMS-amended microcosms although there was a lag phase before methanogenesis started (Fig. 2). Total methane generation was significantly higher in sandy Medway and Nadder sediments at 193 ± 1 and 167 ± 2 µmol methane g^−1^ than the gravel sediments from Pant and Rib at 22 ± 1 and 15 ± 0.5 µmol g^−1^ (*p* < 0.05). Stoichiometrically, 1 mol of DMS can be converted to a maximum of 1.5 mol of methane, therefore, these values correspond to methane yields of 83 and 92% in Medway and Nadder, whilst to 40 and 48% in Pant and Rib, respectively. It should also be noted that Medway and Nadder sediments exhibited considerably longer incubation periods (108 and 134 days, respectively) than Pant and Rib sediments (54 days) until methane production reached the peak concentration (Fig. 2A).

CO_2_ production was also measured in the microcosms (Fig. 2B). The highest CO_2_ production occurred in the River Medway microcosms with 95 ± 3 µmol CO_2_ g^−1^ wet sediment. This value was higher than the theoretical CO_2_ yield if DMS is only used through methanogenesis (77 µmol g^−1^, [57, 65]), suggesting an alternative pathway, such as sulfate-reduction, to CO_2_ production in this sediment. On the other hand, CO_2_ concentrations in River Nadder sediment accounted for 57% of the theoretical CO_2_ yield (60 µmol CO_2_ g^−1^). Rivers Pant and Rib had appreciably lower CO_2_ productions at 4.28 ± 0.3 µmol CO_2_ g^−1^ and 2.04 ± 0.2 µmol CO_2_ g^−1^, respectively, corresponding to 12 and 9% of theoretical yields, pointing toward simultaneous CO_2_ consumption in these sediment microcosms.

### Diversity of methanogens in river sediments and DMS-amended microcosms

To identify the methanogens in the sediment microcosms with DMS, the *mcrA* gene was sequenced. A total of 1.4 × 10^6^, quality-filtered, chimaera-free *mcrA* sequences were obtained and assigned to 5240 ASVs.

**Fig. 3:**
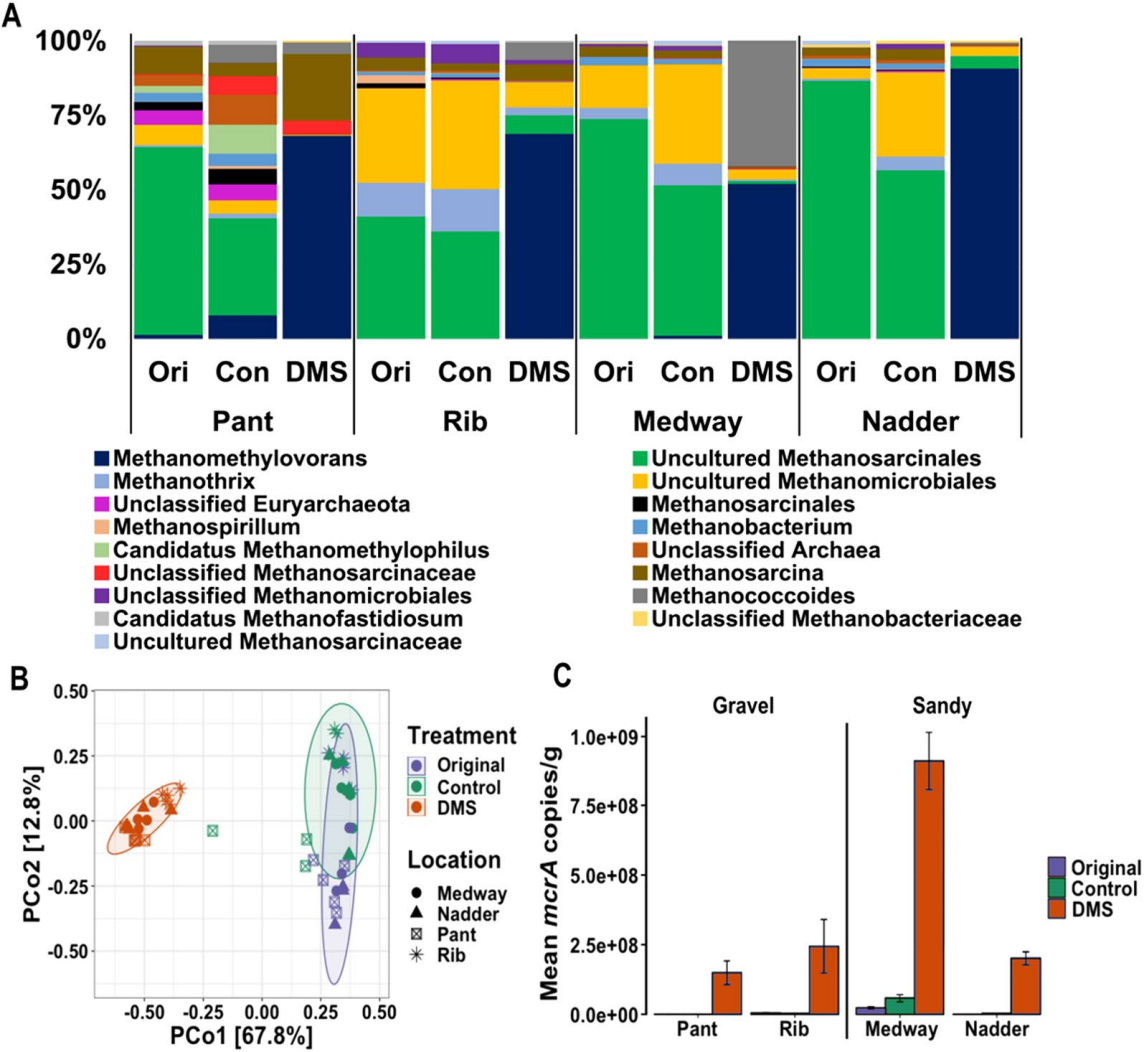
**A** Average relative abundance of the methanogens at the genus level in five original river sediment samples and five replicated DMS microcosms per site as determined via the amplification of the *mcrA* gene. **B** Principal Coordinate Analysis (PCoA) plots of the *mcrA* sequences based on Bray–Curtis dissimilarity metrics. Ellipses indicate 95% confidence intervals according to treatment data. Colours indicate treatment. Shapes indicate sampling sites. **C** Mean copy number of the *mcrA* gene per gram of sediment. Ori: Original sediment samples. Con: Sediment samples from the control microcosms. DMS: Sediment samples from the DMS-amended microcosms. Error bars represent standard error above and below the average of five replicates

The methanogen diversity varied in the original and control sediments and was dominated by uncultured *Methanosarcinales* at relative abundances between 41 and 86%. Uncultured *Methanomicrobiales* (3–36%) and *Methanosarcina* (2–9%) were also observed, while Rib and Medway sediments additionally had *Methanothrix* (4–14%; Fig. 3A). The methanogen diversity shifted significantly when the sediments were incubated with DMS (PERMANOVA, *p* < 0.001), yet no significant difference was observed between the DMS-amended gravel and sandy riverbed sediments (PERMANOVA, *p* > 0.05). This suggests that the community structure of the DMS-degrading methanogens was not affected by the sampling site and grain size. *Methanomethylovorans* became the dominant methanogen genus in the microcosms with relative abundances between 53 and 91%, increasing from ~ 2% in River Pant sediments, ~ 0.4% in River Medway and undetectable levels in the sediments from Rivers Rib and Nadder (Fig. 3A). An increase in the relative abundance of *Methanosarcina* (22 ± 5%) and *Methanococcoides* (42 ± 15%) was also observed in the River Pant and River Medway microcosms, respectively.

The number of methanogens increased in all DMS-amended sediments compared to the original and control sediments (Fig. 3C). River Medway had the highest abundance of in situ methanogens, which also exhibited the highest increase following the DMS addition from 2.3 × 10^7^ ± 0.6 × 10^7^ copies g^−1^ to 9.1 × 10^8^ ± 1.8 × 10^8^ copies g^−1^. The other river sediment DMS microcosms had similar methanogen abundances in the original sediments (0.2 × 10^6^–2.9 × 10^6^ copies g^−1^) and after DMS amendment (1.9 × 10^8^–3.9 × 10^8^ copies g^−1^) despite the difference in sediment grain sizes (Fig. 3C).

Spearman’s r correlation analysis of the PCoA coordinates showed that 71.3% of the factors affecting the methanogen community structure can be attributed to DMS degradation, and methane and CO_2_ production (r_s_ = 0.9, *p* < 0.001; Fig. 3B; Table 1). PCo2 correlated positively with grain size (r_s_ = 0.4, *p* < 0.05), although no significant change was observed in the methanogen diversity between the sediment samples due to the grain size (Table 1).

**Table 1:** Spearman’s rank correlation coefficients (r_s_) between the first two principal coordinates of the *mcrA* diversity at the genus level, the DMS consumed (µmol), methane and CO_2_ produced (µmol) and grain size (mm)

Variable	PCo1	PCo2
DMS consumed (µmol)	0.87***	0.03
Methane produced (µmol)	0.87***	0.02
CO_2_ produced (µmol)	0.86***	0.02
Grain size (mm)	− 0.08	0.43*

Statistically significant values are given in bold

***p < 0.001; *p < 0.05

### Taxonomic analysis of metagenomes and potential metabolic pathways

**Fig. 4:**
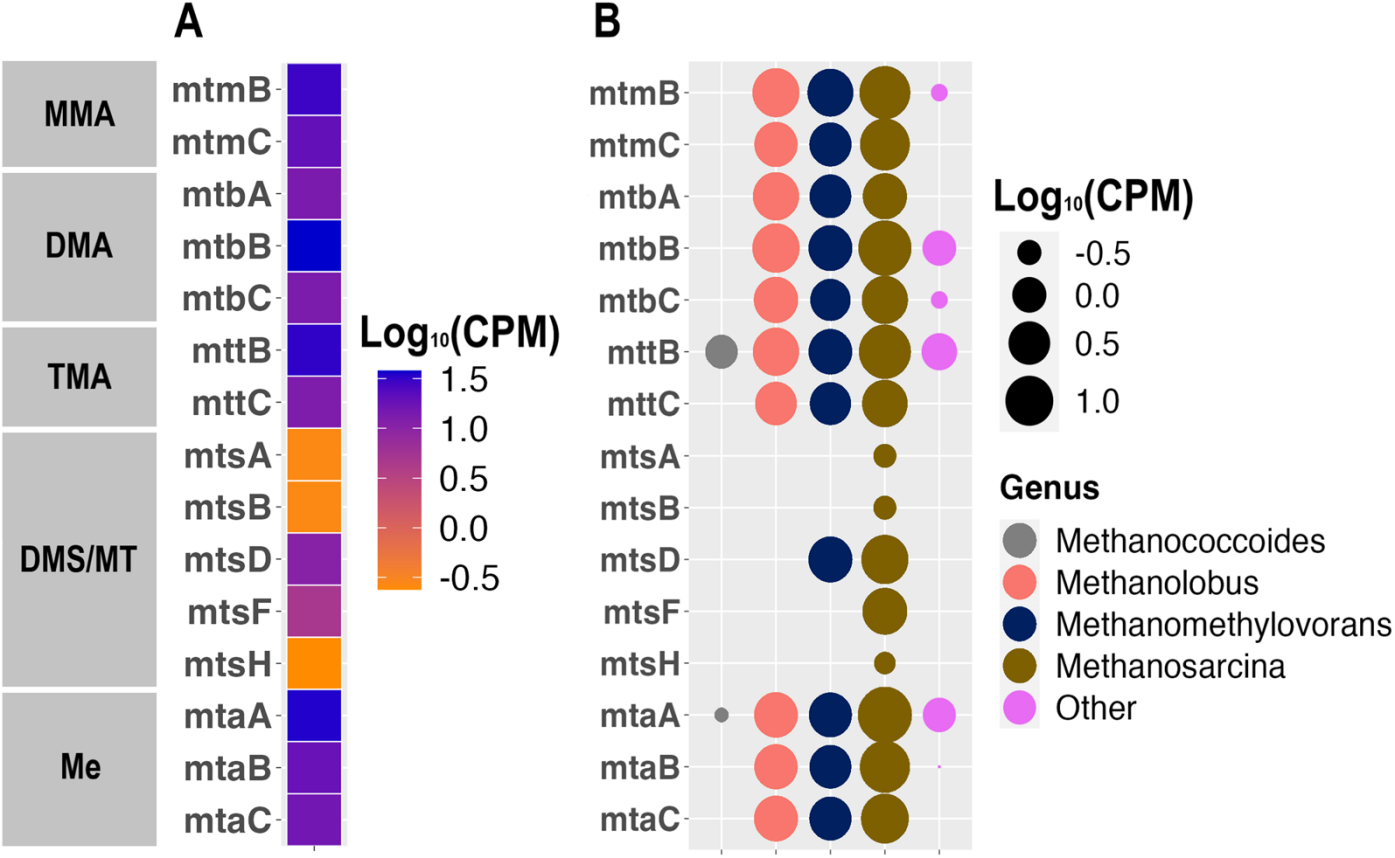
**A** Heatmap showing the abundance of genes involved in methylotrophic methanogenesis in River Pant sediment microcosm with DMS. CPM: Copies per million reads. MMA: Monomethylamine, DMA: Dimethylamine, TMA: Trimethylamine, DMS: Dimethylsulfide, MT: Methanethiol, Me: Methanol **B** Bubble graph showing the abundance of the methanogens affiliated with methylotrophic methanogenesis-related genes in River Pant sediment microcosm with DMS. Other: Genera with a gene copy number < 50

Taxonomic assignment of the metagenomic sequences from the River Pant DMS microcosms showed that *Methanosarcina* and *Methanomethylovorans* dominated the archaeal populations with relative abundances of 50 and 32%, respectively, as was also detected by *mcrA* sequencing. These genera have been reported to include DMS-degrading strains [38, 39, 41, 45]. The *mtsA*, *mtsB* and *mtsH* genes, which were shown to be the key genes in the DMS-dependent methane production in pure cultures of *Methanosarcina barkeri* and *Methanosarcina acetivorans*, had low relative abundances in the metagenomic dataset (0.02, 0.02 and 0.01%, respectively) and belonged to *Methanosarcina* (Fig. 4B,[18, 63]). The *mtsF* and *mtsD* genes were found in comparatively higher relative abundances (0.30 and 0.64%, respectively) and were taxonomically assigned to *Methanosarcina* and *Methanomethylovorans* (Fig. 4A and B). This suggests they are likely involved in DMS degradation in the River Pant sediment. Amongst the other methylotrophic methanogenesis-related genes, the methanol-, trimethylamine- and dimethylamine-specific methyltransferases, *mtaA*, *mttB* and *mtbB*, stand out with the highest relative abundances at 2.1, 2, and 2.3%, respectively (Fig. 4A). These genes were most closely affiliated with *Methanosarcina*, *Methanomethylovorans* and *Methanolobus* (Fig. 4B). The rest of the methylotrophic methanogenesis-related genes (*mtmBC*, *mtbABC*, *mttC*, and *mtaBC*) had 0.8–1.8% relative abundance and were primarily assigned to the genera *Methanosarcina*, *Methanomethylovorans* and *Methanolobus* (Fig. 4B).

All the genes in the *mcrABCDG* operon, which encodes methyl-CoM reductase catalysing the final step in methane formation, had a relative abundance of 0.4% (Supplementary Fig. 1). These *mcr* genes were from the genera *Methanosarcina*, *Methanomethylovorans* and *Methanolobus*. Furthermore, several other genes in central methanogenic pathways (e.g. *mtrA-H*, *hdrABCD*, *mvdADG*, *frhB*) were found, however, the *fpo* and *vho* genes catalysing coenzyme B/coenzyme M regeneration were not retrieved from the metagenome sequences (Supplementary Fig. 1).

A total of eight metagenome-assembled genomes (MAGs) were obtained from the Pant metagenome dataset (seven medium quality and one high quality; Supplementary Table 3). Yet, no methanogen MAG was recovered.

## Discussion

The importance of rivers and streams for global methane emissions has only recently been recognised. Therefore, knowledge of the microorganisms and pathways underlying the production of methane in river sediments is limited.

All the river sediments tested within this study demonstrated considerable potential to produce methane via DMS degradation. The sandy sediments from River Medway and Nadder had the highest DMS-to-methane yields with 83 and 92%. To the best of our knowledge, this is the maximum recorded methane yield from DMS degradation, which highlights the substantial potential of DMS as a methane precursor in sandy, less permeable river sediments. Interestingly, we previously showed a 41% methane yield in River Medway sediment [68]. Higher rainfall and river flow followed by increased aeration and decreased nutrient concentrations were likely the reasons for lower methanogenic activity in our previous study. River Medway received 18 mm of rainfall in November 2018 compared to the 7 mm rainfall in March 2021 when we carried out sampling for the current study [70, 71]. Despite substantial DMS-dependent methanogenesis potential in all river sediments, there was a large difference in yields between the sediments with different grain sizes. The gravel Pant and Rib sediments exhibited methane yields between 40 and 48%, which are lower than those obtained from the sandy riverbed sediments but comparable to a study by Kiene et al. [29] on lake sediment (63%) and our previous study on River Medway (41%). In addition to the permeability differences, sandy river sediments might have received more nutrients and organic material than gravel sediments, which likely led to higher methane yields [44, 60].

*Methanomethylovorans* dominated the DMS-amended sediments from all rivers although the only original samples we detected this genus were from the Rivers Pant and Medway. This points towards DMS-degrading methanogens having low in situ abundances in river sediments tested here. *Methanomethylovorans* are known methylotrophic methanogens, which were shown to degrade DMS in freshwater ecosystems [10, 23, 38, 39, 68]. Our results demonstrated that the grain size and consequently the sediment permeability of river sediments did not affect the diversity of methanogens with DMS degradation potential, however, it strongly affected the capacity of this genus to degrade the available DMS to methane since different methane yields were obtained from different samples. *Methanosarcina* also increased in Rivers Pant and Rib following the addition of DMS. Members of the genus *Methanosarcina* are known to utilise most methanogenic substrates, including DMS [14, 41, 45, 61]. Therefore, *Methanosarcina* species likely performed DMS-dependent methanogenesis in the River Pant and Rib sediments. Similarly, *Methanococcoides,* another methylotrophic methanogen genus, increased in Rivers Pant, Rib and Medway, following the addition of DMS although no member of the *Methanococcoides* genus has been shown to degrade DMS before [21, 33, 34]. Alternatively, they might have performed mixotrophy using CO_2_ produced as a metabolite during DMS degradation as was previously shown for *Methanococcoides methylutens* [78]. *Methanococcoides* are typically associated with saline environments such as saltmarshes, brackish lakes and tidal flats [43, 69, 75]. A plausible way for *Methanococcoides* to be introduced to River Pant, Rib and Medway sediments is via runoff from the surrounding terrestrial soils, where these methanogens have previously been found [72]. In River Medway, *Methanococcoides* have likely been transported from the marine sediments via tidal mixing since this river is part of the macrotidal Medway Estuary [68].

We analysed the key functional genes of methylotrophic methanogenesis in the metagenomics datasets and found that the *mtsA, mtsB, mtsF* and *mtsH* genes had the lowest abundance despite the Mts enzyme having been recognised as the key DMS methyltransferase [18, 63, 64]. *mtsD* was the only *mts* gene found in relatively high abundance (0.64% compared to < 0.02%) and was likely involved in DMS-dependent methanogenesis in the river sediments tested. On the other hand, the *mtaA*, *mttB* and *mtbB* were found at high relative abundances (2.1–2.3%), suggesting that DMS-degrading methanogens in our samples used methanol-, trimethylamine- or dimethylamine-methyltransferase to transfer the methyl group from DMS to Co-M. This is similar to our recent study, where, using metagenomics and metatranscriptomics, we showed that *Methanolobus* likely used trimethylamine and methanol methyltransferases to degrade DMS in Baltic Sea sediments [67]. Alternatively, there may be currently unknown genes or pathways of DMS-dependent methanogenesis.

## Conclusion

This was the first study investigating methane production via DMS degradation in river sediments with contrasting riverbed geologies. Our results highlight the previously overlooked potential of DMS as a methane precursor in river ecosystems particularly those with sandy sediments. *Methanomethylovorans* were the dominant DMS-degrading methanogens in all sampling sites regardless of the gravel size, highlighting a significant role for this genus in riverine methane production. Hence, the contribution of DMS to methane production in river sediments and the response of *Methanomethylovorans* to changing climatic conditions warrant further study to better understand the methane production pathways in rivers and predict the future global methane budget.

### Supplementary Information

Supplementary Material 1

## Data Availability

All sequence data produced in this study are publicly available. The *mcrA* gene sequences are deposited at the NCBI Sequence Read Archive with the BioProject ID PRJNA1083105. The metagenomics dataset is available in the JGI GOLD database (Project ID: Gp0507774). All scripts for figure creation and sequence analysis are available on Github and Zenodo (Tsola 2024, 10.5281/zenodo.10775199).
