# Supplementary material for: Methanomethylovorans are the dominant dimethylsulfide-degrading methanogens in gravel and sandy river sediment microcosms
Source: Environ Microbiome. 2024 Jul 20;19:51. doi: 10.1186/s40793-024-00591-4 (PMC11264916; doi:10.1186/s40793-024-00591-4)
Supplement: Supplementary file 1 — Supplementary Material 1 [file 40793_2024_591_MOESM1_ESM.pdf]

Methanomethylovorans are the dominant dimethylsulfide-degrading methanogens in gravel and sandy riverbed sediment microcosms

S. L. Tsola[1], A. A. Prevodnik[1], L. F. Sinclair[1], I. A. Sanders[1], C. K. Economou[1], and Ö. Eyice[1*]

[1] School of Biological and Behavioural Sciences, Queen Mary University of London, London, UK

[*] For correspondence. E-mail: o.eyice@qmul.ac.uk

Supplementary Material

Supplementary Table 1. Grain size characterisation of the four rivers. Rivers Pant and Rib have gravel-dominated riverbeds. Rivers Medway and Nadder have sand-dominated riverbeds.

River	Gravel (%)	Sand (%)
Pant	**66.7**	33.3
Rib	**59.8**	40.2
Medway	14.0	**86.0**
Nadder	9.9	**90.1**

Supplementary Table 2. The list of 52 methanogenesis-related genes searched within the Pant metagenome dataset.

mtmB	Methanogenesis (Methylamine)	methylamine---corrinoid protein Co-methyltransferase	K16176
mtmC	Methanogenesis (Methylamine)	monomethylamine corrinoid protein	K16177
mtbA	Methanogenesis (Dimethylamine)	[methyl-Co(III) methylamine-specific corrinoid protein]:coenzyme M methyltransferase	K14082
mtbB	Methanogenesis (Dimethylamine)	dimethylamine---corrinoid protein Co-methyltransferase	K16178
mtbC	Methanogenesis (Trimethylamine)	dimethylamine corrinoid protein	K16179
mttB	Methanogenesis (Trimethylamine)	trimethylamine---corrinoid protein Co-methyltransferase	K14083
mttC	Methanogenesis (Trimethylamine)	trimethylamine corrinoid protein	K14084
mtsA	Methanogenesis (Dimethylsulfide, methanethiol, methylpropanoate)	methylthiol:coenzyme M methyltransferase	K16954
mtsB	Methanogenesis (Dimethylsulfide, methanethiol, methylpropanoate)	methylated-thiol--corrinoid protein	K16955
mtsD	Methanogenesis (Dimethylsulfide, methanethiol, methylpropanoate)	methyltransferase cognate corrinoid protein [Methanosarcina acetivorans C2A]	MA0859
mtsF	Methanogenesis (Dimethylsulfide, methanethiol, methylpropanoate)	cobalamin-dependent protein [Methanosarcina acetivorans C2A]	MA4384
mtsH	Methanogenesis (Dimethylsulfide, methanethiol, methylpropanoate)	cobalamin-dependent protein [Methanosarcina acetivorans C2A]	MA4558
mtaA	Methanogenesis (Methanol)	[methyl-Co(III) methanol/glycine betaine-specific corrinoid protein]:coenzyme M methyltransferase	K14080
mtaB	Methanogenesis (Methanol)	methanol---5-hydroxybenzimidazolylcobamide Co-methyltransferase	K04480
mtaC	Methanogenesis (Methanol)	methanol corrinoid protein	K14081
mcrA	Coenzyme M reduction to methane	methyl-coenzyme M reductase alpha subunit	K00399
mcrB	Coenzyme M reduction to methane	methyl-coenzyme M reductase beta subunit	K00401
mcrC	Coenzyme M reduction to methane	methyl-coenzyme M reductase subunit C	K03421
mcrD	Coenzyme M reduction to methane	methyl-coenzyme M reductase subunit D	K03422
mcrG	Coenzyme M reduction to methane	methyl-coenzyme M reductase subunit gamma	K00402
cooF	Coenzyme B/Coenzyme M regeneration	anaerobic carbon-monoxide dehydrogenase iron sulfur subunit	K00196
cooS	Coenzyme B/Coenzyme M regeneration	anaerobic carbon-monoxide dehydrogenase catalytic subunit	K00198
fdhA	Coenzyme B/Coenzyme M regeneration	glutathione-independent formaldehyde dehydrogenase	K00148
fdhB	Coenzyme B/Coenzyme M regeneration	formate dehydrogenase (coenzyme F420) beta subunit	K00125
fpoA	Coenzyme B/Coenzyme M regeneration	F420H2 dehydrogenase subunit A	K22158
fpoB	Coenzyme B/Coenzyme M regeneration	F420H2 dehydrogenase subunit B	K22159
fpoC	Coenzyme B/Coenzyme M regeneration	F420H2 dehydrogenase subunit C	K22160
fpoD	Coenzyme B/Coenzyme M regeneration	F420H2 dehydrogenase subunit D	K22161
fpoF	Coenzyme B/Coenzyme M regeneration	F420H2 dehydrogenase subunit F	K22162
fpoH	Coenzyme B/Coenzyme M regeneration	F420H2 dehydrogenase subunit H	K22163
fpoI	Coenzyme B/Coenzyme M regeneration	F420H2 dehydrogenase subunit I	K22164
fpoJ	Coenzyme B/Coenzyme M regeneration	F420H2 dehydrogenase subunit J	K22165
fpoK	Coenzyme B/Coenzyme M regeneration	F420H2 dehydrogenase subunit K	K22166
fpoL	Coenzyme B/Coenzyme M regeneration	F420H2 dehydrogenase subunit L	K22167
fpoM	Coenzyme B/Coenzyme M regeneration	F420H2 dehydrogenase subunit M	K22168
fpoN	Coenzyme B/Coenzyme M regeneration	F420H2 dehydrogenase subunit N	K22169
fpoO	Coenzyme B/Coenzyme M regeneration	F420H2 dehydrogenase subunit O	K22170
frhA	Coenzyme B/Coenzyme M regeneration	coenzyme F420 hydrogenase subunit alpha	K00440
frhB	Coenzyme B/Coenzyme M regeneration	coenzyme F420 hydrogenase subunit beta	K00441
frhD	Coenzyme B/Coenzyme M regeneration	coenzyme F420 hydrogenase subunit delta	K00442
frhG	Coenzyme B/Coenzyme M regeneration	coenzyme F420 hydrogenase subunit gamma	K00443
hdrA	Coenzyme B/Coenzyme M regeneration	heterodisulfide reductase	K03388
hdrB	Coenzyme B/Coenzyme M regeneration	heterodisulfide reductase	K03389
hdrC	Coenzyme B/Coenzyme M regeneration	heterodisulfide reductase	K03390
hdrD	Coenzyme B/Coenzyme M regeneration	heterodisulfide reductase	K08264
hdrE	Coenzyme B/Coenzyme M regeneration	heterodisulfide reductase	K08265
mvdA	Coenzyme B/Coenzyme M regeneration	F420-non-reducing hydrogenase large subunit	K14126
mvdD	Coenzyme B/Coenzyme M regeneration	F420-non-reducing hydrogenase iron-sulfur subunit	K14127
mvdG	Coenzyme B/Coenzyme M regeneration	F420-non-reducing hydrogenase small subunit	K14128
vhoA	Coenzyme B/Coenzyme M regeneration	methanophenazine hydrogenase, large subunit	K14068
vhoC	Coenzyme B/Coenzyme M regeneration	methanophenazine hydrogenase, cytochrome b subunit	K14069
vhoG	Coenzyme B/Coenzyme M regeneration	methanophenazine hydrogenase	K14070

Supplementary Table 3. Metagenome assembled genomes (MAGs) constructed from the Pant metagenome datasets. Quality is based on the MIMAG (Bowers et al., 2017). Comp: Completeness; Cont: Contamination.

Kingdom	Organism	Comp	Cont	Bases	Genes	Quality
Bacteria	UBA9579 (Gastranaerophilales)	95.73%	4.27%	2,595,968	2,568	HQ
Bacteria	LSR1 (Thiobacillaceae)	97.63%	0.70%	3,455,903	3,364	MQ
Bacteria	Sulfuricurvum (Sulfurimonadaceae)	99.59%	0.41%	2,185,547	2,264	MQ
Bacteria	NBLH01 (Bacteroidales)	73.75%	4.84%	4,285,354	4,068	MQ
Bacteria	UBA9959 (Elusimicrobiales)	54.42%	0%	1,399,045	1,536	MQ
Bacteria	UBA12480 (Delongbacteria)	97.80%	1.10%	2,772,328	2,499	MQ
Bacteria	Micavibrionales_A (Alphaproteobacteria)	86.90%	2.84%	2,250,259	2,210	MQ
Bacteria	UBA6107 (Anaerolineaceae)	58.15%	2.48%	1,555,370	1,668	MQ

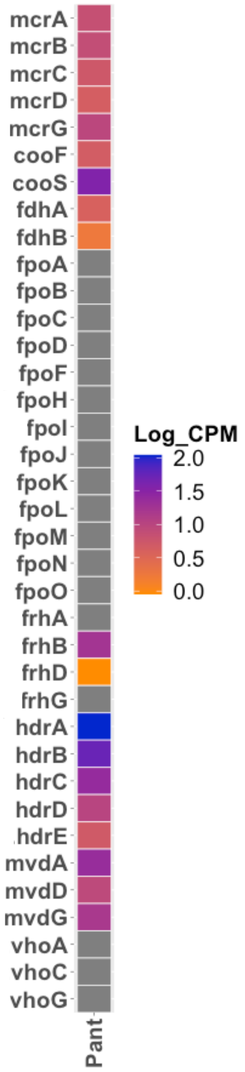

Supplementary Figure 1. Heatmap showing the normalised copy numbers of the genes common in all methanogenesis pathways. CPM: Copies per million reads.
